# Effect of infant feeding on maternal body composition

**DOI:** 10.1186/1746-4358-3-18

**Published:** 2008-08-06

**Authors:** Irene E Hatsu, Dawn M McDougald, Alex K Anderson

**Affiliations:** 1 District 2 Public Health, Gainesville, GA, USA; 2 Department of Foods and Nutrition, University of Georgia, Athens, GA, USA

## Abstract

**Background:**

Women gain total body weight and accrue body fat during pregnancy. Breastfeeding has been suggested as an efficient means of promoting postpartum weight loss due to its high energy cost. We investigated the effect of infant feeding mode on maternal body composition.

**Methods:**

This study evaluated maternal weight and percent body fat changes in exclusively breastfeeding versus mixed feeding mothers during the first 12 weeks postpartum using the BOD POD. Twenty four mothers aged 19 – 42 years were studied. Participants were recruited from Athens-Clarke County and surrounding areas of the State of Georgia, USA. The study was conducted between November 2005 and December 2006.

**Results:**

Prepregnancy weight was higher in mixed feeding mothers than in exclusively breastfeeding mothers (68.4 kg vs. 61.4 kg) but the difference was not statistically significant. At 12 weeks postpartum, exclusively breastfeeding mothers had lost more total body weight than mixed feeding mothers (4.41 ± 4.10 kg versus 2.79 ± 3.09 kg; p = 0.072). There was no significant difference in fat weight change between the two groups (4.38 ± 2.06 kg versus 4.17 ± 2.63 kg). However, mixed feeding mothers lost slightly more percent body fat than exclusively breastfeeding mothers (1.90 ± 4.18 kg versus 1.71 ± 3.48 kg), but the difference was not statistically significant. The trend in percent body fat loss was significant among exclusively breastfeeding mothers (p = 0.034) but not mixed feeding mothers (p = 0.081). Exclusively breastfeeding mothers consumed more calories than mixed feeding mothers (1980 ± 618 kcal versus 1541 ± 196 kcal p = 0.08). Physical activity levels were, however, higher in mixed feeding mothers than exclusively breastfeeding mothers.

**Conclusion:**

Our results provide further evidence that exclusive breastfeeding promotes greater weight loss than mixed feeding among mothers even in the early postpartum period. This suggests that there is the need to encourage mothers to exclusively breastfeed as a means of overweight and obesity prevention.

## Background

During pregnancy, women gain total body weight and accrue body fat. These body composition changes often last into the postpartum period, and thus can create significant concern for mothers who are eager to return to their prepregnancy weight. Lactation is often suggested by researchers as an efficient means of postpartum weight loss due to its high energy cost [1-5]. The literature indicates that findings from studies evaluating the effect of lactation on postpartum weight retention/loss are mixed. The mixed findings could be attributed to variations in study design and methods used to measure total weight or body composition. Errors associated with most of the traditional methods used in total weight (self-reported or on-site measurement) or body composition measurements could also be a reason why the results of the studies in this area are inconclusive [1-17].

The BOD POD, which uses the air-displacement plethysmography technique, is an accurate, non-invasive piece of equipment invented in the mid 1990s to measure body composition. It has a brief measurement period which enables multiple readings over a short period of time. Despite its accuracy, the BOD POD has not previously been used to assess the effect of infant feeding on postpartum body composition changes. To fill this gap in infant feeding and body composition research, the BOD POD was used to evaluate the effect of infant feeding on postpartum maternal body composition. We hypothesized that mothers who exclusively breastfeed their newborns would experience greater postpartum weight loss and percent body fat loss than their counterparts who mixed feed. We believe the results from such a study will help to further understand the impact of infant feeding on maternal body composition. In this paper we present data on maternal postpartum weight changes, percent body fat changes, dietary intake and physical activity.

## Methods

### Study design

This was a longitudinal study conducted in Athens-Clarke County and surrounding counties of the State of Georgia between November 2005 and December 2006. Participants recruited from the above mentioned areas reported to the Maternal and Child Nutrition Research Lab (MCNRL), Department of Foods and Nutrition, at The University of Georgia (UGA), USA, for interviews and body composition measurements. Participants were interviewed using a structured questionnaire containing both closed- and open-ended questions, which was followed by body composition measurements.

Immediately after the interview, the participant changed into a swimsuit for the body composition measurement. Height and weight were measured before entering the BOD POD for the body composition measurement. The study involved five testing days over a period of four months (36 weeks pregnancy, 2, 4, 8, and 12 weeks postpartum). All participants gave written consent before being recruited into the study after being thoroughly informed of the purpose, requirements and procedures of the study. They were also given the opportunity to read the informed consent form. The study protocol and consent form were approved by the Human Subjects Institutional Review Boards (IRBs) of the University of Georgia (UGA) and the Athens Regional Medical Center (ARMC).

Research participants were recruited via newspaper advertisements, flyers posted throughout the UGA campus, mailings to specific listservs, study brochures at doctors' offices and by word of mouth. Participants were screened for the study inclusion and exclusion criteria.

The primary outcomes were postpartum maternal weight and percent body fat changes, but we also assessed dietary intake and physical activity levels. Sample size was based on the detection of differences in postpartum maternal weight and percent body fat changes among two groups (exclusive breastfeeding and formula feeding) equivalent to 1% body fat. With a type 1 error of 0.05 and a 0.8 probability of detecting a true difference (1 – β), the required sample size per group was 8 [18]. However, because many participating mothers who were formula feeding also gave some breast milk, we ended up with exclusively breastfeeding and mixed feeding groups instead of a formula feeding group.

### Eligibility and exclusion criteria

To be eligible for participation, a participant had to be at least 18 years of age, in their third trimester and not less than 36 weeks pregnant. They had to be non-smokers and not on medication which could affect their body composition such as steroids. Exclusion criteria included complications during pregnancy and delivery, a diagnosis of hypertension, pre-eclampsia or gestational diabetes, as well as pre-term delivery (≤ 37 weeks).

### Study implementation and questionnaires

We collected baseline data at the time of recruitment and follow-up data at 2, 4, 8, and 12 weeks postpartum. All interviews and measurements took place in the MCNRL. During each evaluation, interviews were conducted using both closed and open – ended questionnaires to collect information on health history, infant feeding practice, and physical activity – the type of exercise, its intensity and frequency. Physical activity level was assessed based on the recommendations of the 2005 Dietary Guidelines for Americans [19]. A score for physical activity was derived by multiplying the number of days one exercised within a week by the duration of the activity [20]. For example, a participant who exercised 30 minutes a day, 3 times a week would have an activity score of 1.5 (3 × 0.5). Sedentary or inactive was defined as having an activity score of 0 – 1.4, moderately active was 1.5 – 2.9 and very active was > 3.0. Information was also gathered on dietary habits. On each visit we performed a 24 hour (24-h) dietary recall and calculated the energy and nutrient intake from foods consumed. We also used a food frequency questionnaire (FFQ) alongside the 24-h dietary recall to assess general food intake over a month period. With the 24-h dietary recall, participants were asked to recall every food they ate from morning until evening of the day before evaluation. The recall also included information on portion size as well as brand name and preparation method for each food. Overall energy intake, major macro- and micro-nutrient intakes were assessed using the Nutrition Data System for Research software. Intake of alcohol and dietary fiber were also assessed. The FFQ asked questions about specific foods and how often they were consumed (daily, weekly or monthly), and this information was used as a validation of the information collected by the 24-h dietary recall.

### Body composition measurement

Body composition measurements were conducted using the BOD POD body composition system version 2.30 (Life Measurement Inc, Concord, CA). Participant height was measured to the nearest 1.0 mm using Seca 214 portable stadiometer (Itin Scale Inc, Brooklyn NY) and done in duplicates. Participant weight was measured wearing minimal clothing (bathing suit) and without shoes or jewellery on the body. Weight was measured to the nearest 0.01 kg using a calibrated electronic scale connected to the BOD POD system. Weight measurement was done during the 2-point calibration procedure for the BOD POD, described elsewhere [21].

Once these measurements were done, the computer calculated the corrected body volume (Vb_corr_) which was used in calculating body density (Db) [22]. The BOD POD software then calculated the percent body fat using the Siri equation [23] for Caucasians and the general population or the Ortiz equation [24] for African Americans.

### Outcome variables

The main outcome variables were changes in maternal total body weight and percent body fat. These were assessed at each evaluation time point. Changes in weight and percent body fat loss were calculated by subtracting weight or percent body fat at 2 weeks postpartum from that recorded at each evaluation time point.

### Independent variables

Different one-time covariates were assessed at 36 weeks pregnancy and others at 2 weeks postpartum. These included educational background, delivery type, parity, marital status, age, pregnancy weight gain, and ethnicity. A number of time varying covariates were also assessed at each evaluation time point. These included dietary/energy intake, physical activity and infant feeding practice. The categories of infant feeding investigated in this study were exclusively breastfeeding and mixed feeding. Exclusive breastfeeding was defined as the "infant receiving only breast milk; no other liquid or solid is fed." Mixed feeding was defined as the "infant receiving some breast milk and formula as well as some solids whatever the case may be".

### Statistical analysis

We performed all data entry and analyses using SPSS for Windows version 15.0 (SPSS Inc, Chicago, IL). Dietary information was analyzed using the Nutrition Data System for Research software (Nutrition Coordinating Center, University of Minnesota School of Public Health). Differences in variables by group were evaluated using *t *tests and analysis of variance (ANOVA) for continuous variables, and chi-square tests for categorical variables. Chi-square analyses were used to examine bivariate associations between the outcome variable and independent variables. Since the study has a longitudinal design with repeated measures over time, we conducted repeated measures analysis to assess trends in body composition changes. Multiple linear regression analysis was conducted to identify the relationship between maternal characteristics and the changes in maternal percent body fat over time. Data are reported as means ± SD. P value of ≤ 0.05 was used as a criterion for reporting statistical significance.

## Results

### Participant characteristics

Of the 35 participants we recruited for the study, 11 were lost to follow-up (5 to voluntary withdrawal and 6 to complications from the pregnancy and delivery). Some of the complications reported were pre-eclampsia (n = 1), participant being put on bed rest (n = 3), having a baby with a heart condition in utero (n = 1), and having blood transfusion during delivery (n = 1). Twenty four participants completed the 12 week postpartum follow-up. There were no significant differences in characteristics between participants who were lost to follow-up and those who completed the 12 weeks of follow-up. Table 1 shows the characteristics of the remaining 24 participants who successfully completed the study and were included in all analyses. Seventeen participants were exclusively breastfeeding (EBF) while the other seven were classified as mixed feeding (MF) through the 12 weeks of follow-up. All seven mothers in the MF group introduced infant formula on day 1 after delivery in addition to breast milk, except one mother who did not give any breast milk to her newborn throughout the 12 weeks of the study. At baseline, the groups did not differ significantly in demographic characteristics (Table 1). Average age for participants was 29.6 ± 5.7 (range: 19 – 42) years. Average height for participants was 164.1 ± 6.8 cm. Eighty three percent of the participants had some college education or more, with the average number of years of schooling being 17.1 ± 2.4 years. Eighty eight percent of participants were married, 79% were Caucasians and 21% were Black, non-Hispanics. Forty six percent of the participants were primiparous. The most common delivery method among participants was vaginal delivery (92%) (Table 1).

**Table 1:** Participant demographic characteristics*

**Variables**	**Exclusive breastfeeding** **(n = 17)** ** Mean ± SD**	**Mixed feeding** **(n = 7)** ** Mean ± SD**
Age (yrs)	30 ± 6.0	29 ± 5.1
Education (yrs)	17 ± 2.2	18 ± 2.9
	% (n)	% (n)
Ethnicity		
White	77 (13)	86 (6)
Black	24 (4)	14 (1)
Marital Status		
Married	82 (14)	100 (7)
Single	18 (3)	0 (0)
Parity		
Primiparous	47 (8)	43 (3)
Multiparous	53 (9)	57 (4)
Type of delivery		
Vaginal/spontaneous	94 (16)	86 (6)
Cesarean	6 (1)	14 (1)

*Differences between the groups were not statistically significant.

### Prepregnancy and postpartum body weight changes

**Figure 1:**
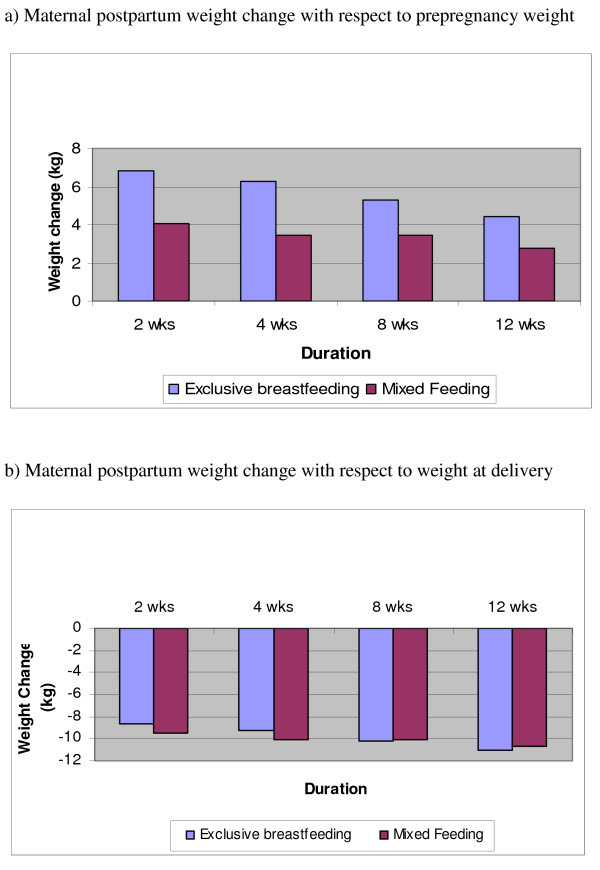
**a – Maternal postpartum weight change with respect to prepregnancy weight.** b – Maternal postpartum weight change with respect to weight at delivery.

**Figure 2:**
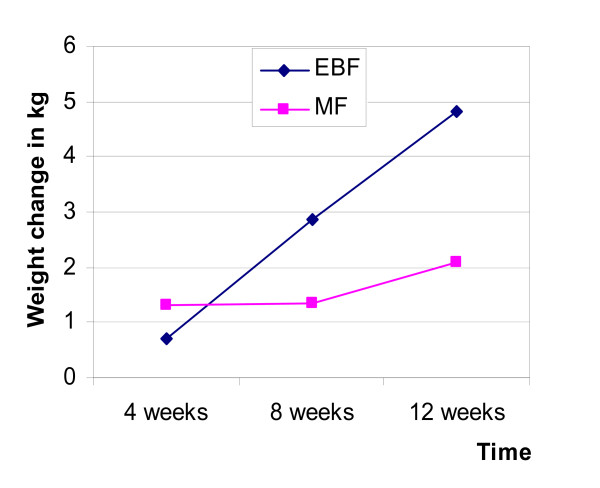
**Pattern of postpartum maternal weight loss by feeding group**. EBF = exclusive breastfeeding, MF = mixed feeding.

Mean self-reported prepregnancy weight for study participants was 63.4 ± 10.9 kg. MF mothers weighed more than EBF mothers (68.4 kg vs. 61.4 kg; p = 0.159) and also had higher BMIs (25.9 kg/m^2 ^vs. 22.1 kg/m^2^; p = 0.04). There was no significant difference in infant birth weight between the two groups (Table 2). Weight change among the two groups was compared with respect to self-reported prepregnancy weight and total weight at delivery. Although there was weight loss within both groups, the weight loss with respect to prepregnancy weight within the EBF group was significantly (p < 0.05) and consistently more at all time points than in the MF group (Figure 1a). There was no significant difference in weight loss both within and between groups with weight at delivery as the reference (Figure 1b). With respect to maternal weight at 2 weeks postpartum, the rate and amount of weight loss was greater for EBF than MF mothers throughout the duration of the study except at 4 weeks when weight loss was higher among MF groups (Figure 2; p = 0.072). Although, the difference in weight loss between the two groups was not significantly different, the magnitude of weight loss was greater among EBF mothers than MF mothers.

**Table 2:** Participant anthropometrics*

	**Exclusive breastfeeding** **(n = 17)** ** Mean ± SD**	**Mixed feeding** **(n = 7)** ** Mean ± SD**
Prepregnancy weight (kg)	61.4 ± 8.1	68.4 ± 15.6
Height (cm)	165.8 ± 6.6	160.0 ± 6.0
Prepregnancy BMI (kgm^-2^)	22.1 ± 2.8	25.9 ± 6.2
Weight at 36 wks pregnancy (kg)	75.6 ± 9.6	81.1 ± 13.2
Pregnancy weight gain (kg)	14.8 ± 4.2	13.7 ± 3.3
Delivery weight (kg)	76.8 ± 9.8	81.9 ± 13.6
Infant birthweight (kg)	3.4 ± 0.3	3.3 ± 0.4

*Differences between the groups were not statistically significant.

### Changes in body composition

Regarding changes in body composition with respect to measurement at 2 weeks postpartum, both groups gained some fat free mass at 4 weeks. The gain in fat free mass was slightly higher among MF compared to EBF mothers, even though the difference was not statistically significant (0.88 ± 1.9 kg vs. 0.28 ± 1.2 kg; p = 0.3) (Table 3). By 8 weeks both groups had lost some fat free mass with respect to measurements at 2 weeks postpartum with EBF mothers losing more than MF mothers (p = 0.09). There was a reverse in the loss of fat free mass at 12 weeks postpartum in both groups with MF mothers gaining significantly more fat free mass than EBF mothers (p = 0.04) (Table 3).

**Table 3:** Lean weight change with respect to lean weight at 2 weeks postpartum

	**Weight change (kg)** ** (Mean ± SD)**		
	
	4 weeks	8 weeks	12 weeks
Exclusive breastfeeding	0.28 ± 1.24	-0.52 ± 1.32	-0.39 ± 1.38
Mixed feeding	0.88 ± 1.89	0.49 ± 1.17	0.99 ± 1.58
Total	0.46 ± 1.45	0.22 ± 1.34	0.01± 1.55

**Figure 3:**
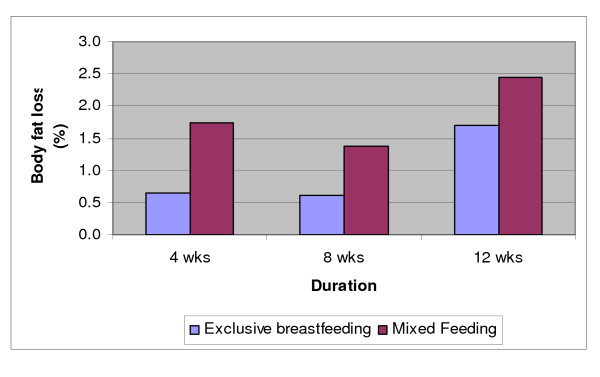
**Maternal percent body fat loss with respect to body fat at 2 weeks postpartum**.

Even though the percent body fat loss was higher in MF than EBF mothers, our repeated measures analysis showed that the rate and trend of percent body fat loss across time was statistically significant for EBF mothers (p = 0.011) but not for MF mothers (p = 0.082) (Figure 3). A similar trend was found for fat mass.

### Dietary intake and physical activity

EBF mothers consumed more calories than MF mothers (1980 ± 618 kcal vs. 1541 ± 196 kcal p = 0.08). They also tended to consume more fat, protein, carbohydrates, dietary fiber, calcium, iron and vitamin D than their MF counterparts (data not shown). Alcohol consumption, however, was higher among MF mothers than EBF mothers (4.8 ± 10.2 g vs. 2.06 ± 4.4 g; p = 0.3) although the difference was not statistically significant. Physical activity assessed based on the 2005 Dietary Guidelines for Americans showed MF mothers exercised more than their EBF counterparts (1.8 ± 0.7 vs. 1.0 ± 0.3). Exercise activities engaged in during pregnancy included aerobics, biking, running, walking, weight-lifting, walking on treadmill and yoga. Participants' engagement in physical activity increased with advancing postpartum period. The most common activity participants engaged in was walking. About 33% of participants engaged in walking at 36 weeks, 22% at 2 weeks, and 38%, 42% and 38% at 4, 8, and 12 weeks, respectively. Other common activities were running/jogging (17%) and aerobics (13%).

### Regression analysis

Since maternal percent body fat is not influenced by a single maternal characteristic, multiple regression analysis was used to assess the independent effects of a number of maternal characteristics as shown in Table 4. The independent variables used in the multivariate regression analysis are prepregnancy BMI, average energy intake, and type of feeding. The standardized regression coefficients show that mothers with higher BMI lost more percent body fat during the postpartum follow-up compared to mothers with lower prepregnancy BMI. Regarding maternal energy intake and postpartum percent body fat changes, the results suggest that mothers who consumed more total energy lost less percent body fat. The regression analysis also showed an inverse relationship between infant feeding and postpartum percent body fat changes with EBF mothers losing more percent body fat in the early postpartum but this association was not significant. The results from the three regression models show that the individual variables did not have significant independent effect on postpartum percent body fat loss, except for maternal pre-pregnancy BMI which was a significant independent predictor (p = 0.034) at 8 weeks postpartum (Table 4), although, the adjusted R^2^s was significant. The adjusted R^2 ^for the regression analyses at 4, 8 and 12 weeks postpartum ranged from 0.188 – 0.251 (p < 0.05). Together, maternal pre-pregnancy BMI, average energy intake and type of infant feeding explained 18.8–25.1% of the variability in postpartum percent body fat loss.

**Table 4:** Factors affecting maternal percent body fat with respect to percent body fat at 2 weeks postpartum

Time point	Variable	Standardized coefficients	P-value	Adjusted R^2^
4 Weeks	Maternal BMI	-0.282	0.202	0.188*
	Average energy intake	-0.358	0.102	
	Type of feeding	-0.307	0.185	
8 Weeks	Maternal BMI	0.485	0.034	0.237♣
	Average energy intake	-0.086	0.685	
	Type of feeding	-0.426	0.071	
12 Weeks	Maternal BMI	0.376	0.109	0.251^¥^
	Average energy intake	-0.179	0.424	
	Type of feeding	-0.348	0.153	

*p = 0.047; ♣ p = 0.038; ^¥^p = 0.035

Type of feeding was coded as 1 = EBF and 2 = MF

## Discussion

### Infant feeding and weight changes

Findings from this study show that mothers with higher prepregnancy BMI chose to mixed feed their newborns. They also lost more percent body fat during the early postpartum period. We found that infant feeding influenced postpartum maternal weight and body composition changes over time, although, the differences seen between the groups were not statistically significant due to the small sample size and short duration of the study. Our results show that weight loss among EBF mothers tended to be higher and at a faster rate compared to their MF counterparts. This finding is in agreement with the results from other studies [2,4] which have found significant postpartum weight loss in exclusively breastfeeding mothers compared to formula feeding mothers. The greatest differences in weight loss, according to these studies, were detected around 3 months postpartum which is confirmed by this study. The amount of weight loss after delivery has been linked to pregnancy weight gain: the more weight gained during pregnancy, the more weight retention after delivery [2]. We found the opposite of the trend reported by Janney et al. [2]. In the current study, we found mothers who gained more weight during pregnancy lost more after delivery.

### Weight changes and dietary intake

In agreement with the findings of Chou et al., [9] this study found that caloric intake was higher in EBF mothers than MF mothers. Their mean energy intake, however, did not exceed the 2000 kcal recommendation for women between the ages 19–30 years, [25] the age range of most of our participants. The low dietary intake recorded in the groups could be due to underreporting by participants. We feel confident about our dietary data as this involved five 24-hour dietary recalls taken over a 12 week period that included at least the intake of one weekend day. There were no statistically significant differences between reported intakes. We also observed a strong correlation between data from the different 24-hr dietary recalls (r > 0.967) for both within and between groups. Even though the energy spent in breast milk production is estimated to be about 500 kcal/day, the US Institute of Medicine recommends that breastfeeding mothers consume an extra 330 kcal/day to make up for the energy used in breast milk production with the difference of 170 kcal/day coming from stored fat [26]. This therefore brings the daily recommended energy intake for breastfeeding mothers to 2330 kcal. EBF mothers did not meet this recommendation. It will therefore be important to ascertain the dietary/energy intake of these women before pregnancy. This will help evaluate the current Recommended Dietary Allowances (RDAs) to determine whether that is contributing to the obesity epidemic facing the U. S. Although EBF mothers were found to exercise less than their mixed feeding counterparts, their lower activity did not affect their weight loss trend. The result from measuring weight loss with respect to weight prior to pregnancy supports the protective association of breastfeeding with postpartum weight loss reported in other studies [1-4,27]. All of these studies used prepregnancy weight as a reference for weight loss or weight retention after delivery; they did not use other time points such as weight at delivery which was used in this study.

### Infant feeding and body composition changes

In both groups, weight loss was derived more from fat mass than lean mass though EBF mothers lost more lean mass than MF mothers. This supports what has been reported by Chou et al. [9] who used the dual energy X-ray absorptiometry (DXA) and skin-fold measurements to measure and compare postpartum body composition of 20 women who were either exclusively breastfeeding or formula feeding. We found no difference in fat weight loss between the two groups by 12 weeks postpartum. Even though MF mothers seemed to have lost more percent body fat than EBF mothers in the current study, the trend in percent body fat loss was significant over time among EBF mothers compared to MF mothers according to the repeated measures analysis. The MF mothers may have lost more fat mass because they had a higher prepregnancy weight and BMI which may be an indicator of higher percent body fat compared to EBF mothers. The difference may also be due to the high dietary fat consumption and low physical activity rates of EBF mothers. Adequate Intake (AI) of dietary fat for women aged 19 – 30 years based on a 2000 kcal diet is between 25 – 35% [25]. EBF mothers were found to consume more than the recommended intake for fat (Table 4). Multiple regression analysis also showed that more calories consumed led to less percent body fat loss. Most of our participants (EBF and MF mothers) met and/or even exceeded the recommendation for macronutrient intake but not for micronutrient intake. EBF mothers especially consumed very high amounts of dietary fat which supports the finding by Chou et al. [9].

### Effect of other covariates

Different studies have identified several covariates that may affect body composition besides infant feeding. These include maternal age, parity, prepregnancy weight, pregnancy weight gain, dietary intake and physical activity [1,2,4,10,12,13]. In this study, we used multiple regression analysis to identify independent factors that affect maternal percent body fat besides infant feeding methods. Maternal characteristics that were identified to have independent effects included prepregnancy BMI and dietary intake. Identification of energy intake in the current study is consistent with what has been reported by Chou et al. [9]. To the best of our knowledge, none of the previous studies in this area have investigated the effect of BMI on maternal body composition. Regression analysis in this study showed that mothers with higher prepregnancy BMI lost more percent body fat after delivery.

Exercising during pregnancy has been found not to affect birth weight but, rather, to have other physiological benefits such as improved labor [28]. Even though this study did not investigate the physiological benefits of exercising during pregnancy, we found that women who were physically active during pregnancy engaged in exercises recommended by the American College of Obstetricians and Gynecologists [29]. Similar to a previous study by Zhang et al., [30] our participants engaged in activities such as walking, running or jogging and aerobics during pregnancy. About 75% of participants engaged in these exercises even at 36 weeks pregnancy. This percentage reduced to about 21% two weeks after delivery but gradually started increasing by 4 weeks postpartum. At 12 weeks postpartum, none of the new mothers had engaged in any high impact activity such as running.

## Conclusion

Results from this study are of public health importance due to the trends observed in total body weight loss between the different feeding groups. There is an indication of a protective effect of EBF against maternal overweight/obesity and signs of rapid return to prepregnancy weight even in the early postpartum period. The observation that percent body fat loss was significant across time within the EBF mothers and not MF mothers is suggestive of the protective effect of EBF against cardiovascular disease and other chronic health conditions. The study clearly shows the importance of encouraging and supporting mothers to breastfeed exclusively as recommended by the American Academy of Pediatrics and the World Health Organization.

Our study has a number of limitations and therefore the findings should be interpreted with caution. First, the study was designed to compare postpartum maternal weight and percent body fat changes between EBF and formula feeding mothers but we ended up comparing with MF mothers because we realized infants of formula feeding mothers had received some breast milk. Second, the data on prepregnancy weight and weight at delivery were self-reported. It is possible that these self-report may have introduced some inaccuracy into our measures of prepregnant BMI and weight gain during pregnancy. At the same time we feel confident to include these variables in our analysis as we found strong correlations between self-reported prepregnancy weights before and after delivery (r = 0.996) as well as strong correlation between self-reported height and measured height (r = 0.997). Third, our inability to detect significant differences between the groups could be due to the small sample size and the short duration of the study. It is possible that the lack of strong association between the groups was due to lack of statistical power and the overlap of breast milk production between EBF and MF women. Another limitation is the use of self-reported dietary intake. This was minimized by taking dietary recall data at different time points and averaging for our analysis. Participants of this study were highly educated with college or more education and therefore the findings may not be generalizable to less than college educated mothers.

## Competing interests

The authors declare that they have no competing interests.

## Authors' contributions

IEH participated in collection, analysis and interpretation of data, writing of manuscript and final approval of version to be published. DMM participated in collection, and analysis of data, editing of manuscript and final approval of version to be published. AKA participated in study design, analysis and interpretation of data, critical review of manuscript, and final approval of version to be published.
